# Prevalence of generalized anxiety disorder and its related factors among infertile patients in Iran: a cross-sectional study

**DOI:** 10.1186/s12955-018-0956-1

**Published:** 2018-06-19

**Authors:** Reza Omani-Samani, Azadeh Ghaheri, Behnaz Navid, Mahdi Sepidarkish, Saman Maroufizadeh

**Affiliations:** Department of Epidemiology and Reproductive Health, Reproductive Epidemiology Research Center, Royan Institute for Reproductive Biomedicine, ACECR, Tehran, Iran

**Keywords:** Generalized anxiety disorder, Infertility, Prevalence, Iran

## Abstract

**Background:**

Generalized anxiety disorder (GAD) is one of the most prevalent anxiety disorders among infertile patients. This study aimed to determine the prevalence of GAD and its associated factors among infertile patients in Tehran, Iran.

**Methods:**

This cross-sectional study included 1146 infertile patients in a referral fertility center in Tehran, Iran between May and October 2017. GAD was measured using the Generalized Anxiety Disorder-7 (GAD-7) scale. The associations between GAD and demographic/fertility characteristics were estimated using simple and multiple logistic regression with odds ratio (OR) and 95% confidence interval (CI).

**Results:**

The mean total GAD-7 score was 6.61 (SD = 5.32). Using a cut-off value of 10, the prevalence of GAD was 28.3%. In adjusted analysis, female sex (OR = 2.54, 95% CI = 1.88–3.42, *P* < 0.001), low educational level (OR = 1.45, 95% CI = 1.08–1.94, *P* = 0.012), high infertility duration (OR = 1.05, 95% CI = 1.01–1.09, *P* = 0.013), and treatment failure (OR = 1.52, 95% CI = 1.13–2.04, *P* = 0.006) were associated with GAD.

**Conclusions:**

The prevalence of GAD is relatively high in infertile patients. We conclude that all infertile patients should be screened for symptoms of GAD and treated for this disorder as need arises.

## Background

Infertility is characterized by “the failure to establish a clinical pregnancy after 12 months of regular, unprotected sexual intercourse or due to an impairment of a person’s capacity to reproduce either as an individual or with his/her partner.” [1], and it is a disease, which generates disability as an impairment of function [1]. It is also a public health problem affecting 9% of reproductive-aged couples worldwide [2], and can lead to negative psychological consequences and diminished quality of life and well-being [3, 4]. Among psychological disorders, anxiety disorder including generalized anxiety disorder (GAD) is one of the most prevalent disorder in infertile patients. GAD is characterized by “excessive and persistent worrying that is hard to control, causes significant distress or impairment, and occurs on more days than not for at least six months” [5], and epidemiological surveys show that this disorder is more prevalent among clinical sample compared with general population. Despite the importance of GAD and its negative consequences, we still know little about the prevalence of GAD and its associated factors in people with infertility problem.

General-population studies in the US show that GAD have a lifetime prevalence of 5.7%, and 1-year prevalence of 3.1% [6, 7]. A review of epidemiological studies in Europe also find similar prevalence [8]. Epidemiologic surveys in general population show that GAD is more common in women, adults, unemployed people, people of low socioeconomic status, those who are widowed, separated, or divorced [9, 10].

In recent years, the study of GAD has received growing attention in both community and clinical sample. Yet, in the fertility context, there are few published studies on the GAD among people with infertility problem and most of the studies have been performed with relatively small sample size or only in infertile women. On the other hand, some recent studies have shown that psychological distress have negative effect on the infertility treatment outcome. Also, there are few studies in the Middle East area using GAD as a useful instrument for measuring anxiety. Thus, we aimed to determine the prevalence of GAD and associated demographic/infertility factors in a relatively large sample of infertile patients.

## Methods

### Participants and study design

This cross-sectional study was conducted at the Infertility Treatment Center of Royan Institute in Tehran, Iran. This center is one of the largest clinics for infertility treatment in Iran [11]. Infertile couples come to this center, not only from the capital of Iran but also from all around the country.

The data were collected using the convenience sampling method between May and October 2017.

After the evaluation phase of treatment, patients were asked to participate in the study. To be eligible for the study the participants had to meet the following criteria: (a) willingness to participate; (b) age over 18 years; (c) men or women with couple infertility; (d) ability to read, write, and comprehend Persian. In total, 1146 patients (from 1400 patients) agreed to take part and filled out the questionnaires completely (response rate: 81.9%).

### Ethical consideration

Approval to perform the current study was obtained from the Ethics Committee of Royan Institute, Tehran, Iran. All infertile patients were fully informed about the objective of the study, and the confidentiality of the data. Patients were also assured that the data would be used only for the purpose of the research and refusal to participate in the study would not affect their current and future treatments in any way. Each patient gave written informed consent prior to participating in the study.

### Questionnaires

#### Demographic/infertility data

Demographic/clinical data including age, gender, educational level, duration of infertility, cause of infertility, failure of previous treatment, and history of abortion were collected. We selected these variables according to the literature review including high-quality studies in the infertility context [12–15].

#### The 7-item generalized anxiety disorder scale (GAD-7)

The GAD-7 is a brief, 7-item self-administrated scale designed to screen for the presence of GAD and to assess the severity of symptoms based on DSM-IV criteria [16]. This scale asks how often respondents have been affected by anxiety symptoms in the last 2 weeks. Each item is scored on a 4-point Likert scale indicating symptom frequency, ranging from 0 (not at all) to 3 (nearly every day). Total score can range from 0 to 21, with higher scores indicating more severe symptoms of GAD. According to the original validation studies, the total score can then be interpreted as suggesting no/minimal anxiety (0–4), mild (5–9), moderate (10–14), or severe (15–21). A cut off score of 10 is suggested as reflecting a possible diagnosis of GAD. The Persian version of GAD-7 has been shown to have satisfactory psychometric properties in infertile people [17]. The GAD-7 showed high internal consistency in this study (α = 0.882).

### Statistical analysis

Data analyses were done using by IBM SPSS Statistics for Windows, Version 22.0 (IBM Crop., Armonk, NY, USA). Continuous variables were presented as mean ± standard deviation (SD) and categorical variables as numbers (percentage). In this study, GAD-7 score was analyzed as a dichotomous variable (GAD absent and GAD present) based on cut-off value of 10. Simple and multiple logistic regression analysis were applied to examine the association between GAD and demographic/fertility variables. The odds ratio (OR) and 95% confidence interval (CI) were calculated. All statistical tests were two-tailed and level of significance was set at 0.05.

## Results

### Description of participants

**Fig. 1:**
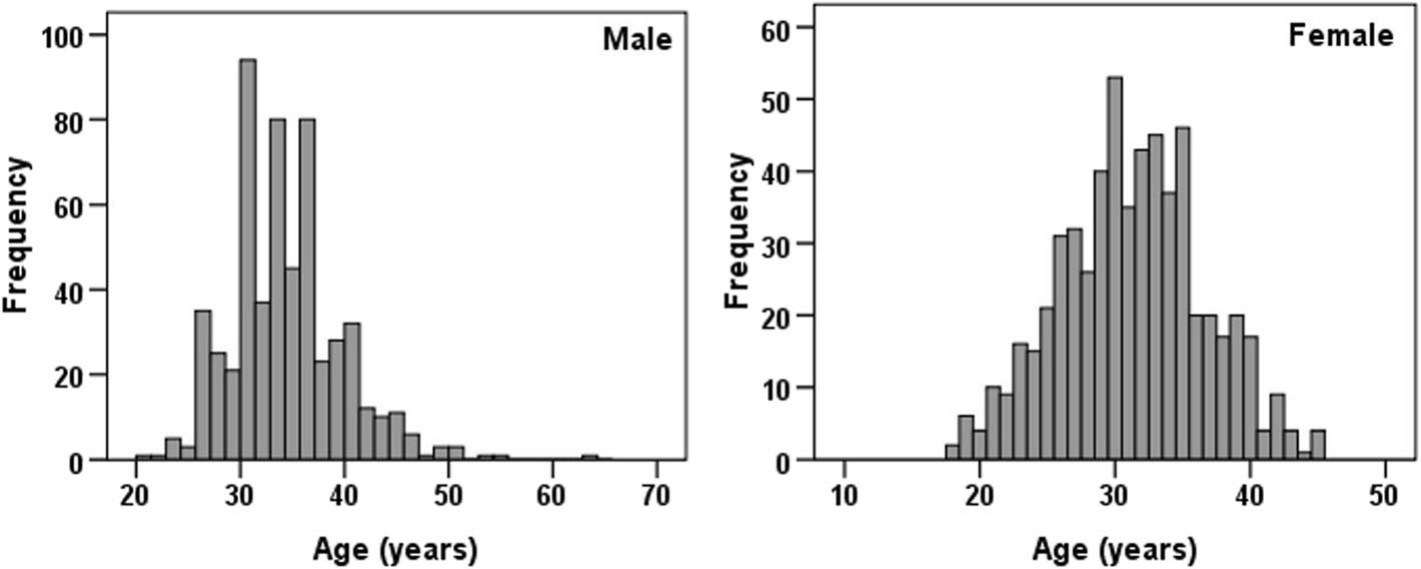
The distribution of age in both men and women

Demographic and fertility information of the participants are summarized in Table 1. The mean (SD) age and infertility duration of the participants were 32.76 (5.54) and 5.40 (4.04) years, respectively. Half (51.2%) were female, 38.1% had male factor infertility, 46.9% were university-educated, 49.7% had at least one failure in previous treatments, and 28.4% had history of abortion. In addition, the distribution of age is shown in Fig. 1.

**Table 1:** Demographic and fertility characteristics of the participants (*n* = 1146)

Variables	Mean ± *SD* or *n* (%)
Age (years)	32.76 ± 5.54
Sex	
Male	559 (48.8)
Female	587 (51.2)
Educational level	
Primary	238 (20.8)
Secondary	370 (32.3)
University	538 (46.9)
Duration of infertility (years)	5.40 ± 4.04
Cause of infertility	
Male factor	437 (38.1)
Female factor	219 (19.1)
Both	208 (18.2)
Unexplained	282 (24.6)
Failure of previous treatment	
No (first treatment)	576 (50.3)
Yes	570 (49.7)
History of abortion	
No	820 (71.6)
Yes	326 (28.4)

*SD*: Standard deviation

### Distribution of GAD-7 score

The mean (SD) total score of GAD-7 was 6.61 (5.32). Distribution of scores falling within GAD-7 severity cut-offs were as follows: no anxiety 43.1%; mild 28.6%; moderate 19.1%; and severe 9.2%. Based on a cut-off value of 10, the prevalence of GAD was 28.3% (*n* = 324).

### Factor associated with GAD

A cut-off value of 10 was used to categorize patients into anxious and non-anxious. Then, simple and multiple logistic regression analysis were used to examine the association between GAD and demographic/fertility variables (Table 2). A cut-off value of 10 was used to categorize patients into anxious and non-anxious. According to adjusted analysis, women were 2.54 times more likely to have GAD than men (OR = 2.54, 95% CI: 1.88–3.42, *P* < 0.001). Primary/secondary-educated individuals were 1.45 times more likely to have GAD than university-educated individual (OR = 1.45, 95% CI: 1.08–1.94, *P* = 0.012). Results showed that each one-year increase in infertility duration increases the odds of being anxious by 5% (OR = 1.05, 95% CI: 1.01–1.09, *P* = 0.013). Participants with partner cause of infertility were less likely to have GAD than those who had self-cause of infertility (OR = 0.76, 95% CI: 0.53–1.09, *P* = 0.134), although this difference was not statistically significant. Finally, logistic regression model showed that patients with at least one failure in their previous treatment were 1.52-fold more likely than others to have GAD (OR = 1.52, 95% CI: 1.13–2.04, *P* = 0.006).

**Table 2:** Association between GAD and demographic/fertility variables among infertile patients

Variables	Simple logistic regression		Multiple logistic regression	
	*OR*_Crude_ (95% *CI*)	*P*	*OR*_Adjusted_ (95% *CI*)	*P*
Age (years)	0.98 (0.96–1.00)	0.063	0.99 (0.96–1.01)	0.340
Sex				
Male	1		1	
Female	2.39 (1.82–3.12)	< 0.001	2.54 (1.88–3.42)	< 0.001
Education				
Primary/Secondary	1.47 (1.13–1.91)	0.004	1.45 (1.08–1.94)	0.012
University	1		1	
Infertility duration (years)	1.07 (1.03–1.10)	< 0.001	1.05 (1.01–1.09)	0.013
Cause of infertility				
Self	1		1	
Partner	1.04 (0.74–1.45)	0.832	0.76 (0.53–1.09)	0.134
Both/Unknown	0.96 (0.70–1.30)	0.779	0.84 (0.61–1.17)	0.312
Treatment failure				
No (first treatment)	1		1	
Yes	1.62 (1.25–2.10)	< 0.001	1.52 (1.13–2.04)	0.006
Previous abortion				
No	1		1	
Yes	1.02 (0.77–1.35)	0.904	0.86 (0.63–1.18)	0.351

CI: Confidence Interval; OR: Odds Ratio

## Discussion

The primary aim of the present study was to determine the prevalence and demographic determinants of GAD among infertile patients in Tehran, Iran using a relatively large data. In this study the prevalence of GAD was 28.3%, which is higher than what was reported in general population [9, 18] and among infertile women in Taiwan (23.2%) [19]. In a study conducted by Maroufizadeh et al. [20] among infertile patients in Iran, the prevalence of overall anxiety using the Hospital Anxiety Depression Scale was 49.6%.

As anticipated, the current study shows that women were 2.54 times more likely to have GAD than man. This finding is consistent with the studies from the general population in which GAD is more common in women than in men [8, 10, 21, 22]. Also, epidemiologic studies in infertile population indicate that anxiety is more prevalent among women than men [13, 23]. Similar results were obtained in previous studies on quality of life and depression among infertile patients [23–25]. These findings suggest that women were more affected by infertility than men in health and psychological status.

Consistent with a study conducted among infertile patients [13], the likelihood of GAD was also increased for infertile patients with low educational level. It seems that higher education brings more information and awareness about the possible negative consequences of infertility, resulting in increased anxiety. In addition, epidemiologic studies indicate that GAD is more prevalent among people of low socioeconomic status (SES) than those of middle or high SES [21].

We found that the odds of GAD increased with rising duration of infertility, which is in line with a great deal of other previous works [20, 23, 26, 27]. In addition, similar trend was reported in other studies on measures of quality of life, marital satisfaction and depression [23, 28, 29]. However, in two studies conducted by Ogawa et al. [30] and Maroufizadeh et al. [13], there was no relationship found between anxiety and duration of infertility.

This study has several limitations that should be mentioned. First, all data are based on self-reports. Second, our measure of GAD may be less valid and reliable than a diagnostic interview.

Third, this study was conducted in one fertility clinic in Tehran; therefore, our results may not be generalizable to other populations. Fourth, the cross-sectional design of the study limits inferences about the causal relationships between GAD and the demographic/fertility variables. Fifth, psychological factors (e.g. social and family support) and some demographic information (e.g. socioeconomic status and type of infertility) that affect GAD were not investigated in this study. Despite these limitations, this study is the first to evaluate the association between GAD and demographic/fertility characteristics among infertile patients in Iran using a relatively large sample size.

## Conclusion

In summary, the prevalence of GAD is relatively high in infertile patients, particularly in women, patients with low educational level and high infertility duration and patients who had failures in the previous infertility treatment. This study suggests that all infertile patients should be screened for symptoms of GAD and treated for this disorder as need arises.

## Abbreviations

CI: Confidence Interval
GAD: Generalized Anxiety Disorder
OR: Odds Ratio
SD: Standard Deviation
SES: Socioeconomic Status
